# An audit of clinical training exposure amongst junior doctors working in Trauma & Orthopaedic Surgery in 101 hospitals in the United Kingdom

**DOI:** 10.1186/s12909-017-1038-5

**Published:** 2018-01-02

**Authors:** BOTA Collaborators, Aaron Rooney, Abdul Nazeer Moideen, Adedeji Akinyooye, Ahmed Fadulemola, Akmal Turaev, Akmal Turaev, Alan Campbell, Alex Mulligan, Alex Goubran, Alexander Durst, Alexander James, Alexander Martin, Alexandra Aframian, Ali Abdelwahab, Ali Abdulkarim, Alisdair Felstead, Alistair Mayne, Aman Sharma, Amanjeet Dahaley, Amit Patel, Amit Thakrar, Anatole Wiik, Andrea Pujol Nicolas, Andrew Hacker, Anna Bridgens, Annie McKirdy, Anoop Prasad, Antonia Hoyle, Ashley Scrimshire, Ashwanth Ramesh, Aurelie Hay-David, Barry Rose, Basil Budair, Bhavin Garara, Blair Tweedie, Cameron Dott, Charlotte Cross, Charlotte Richardson, Christopher Buckle, Christopher Crome, Christopher Ghazala, Christopher Jordan, Claire Coles, Colin Shaw, Conal Quah, Damian Bull, Dan Howgate, Dan Williams, Daniel Burchette, Daniel Shaerf, Daniel Wilson, Danny Ryan, David Butt, David MacDonald, David Milligan, David Neilly, Dev Thakker, Dinnish Baskaran, Donald Hansom, Ed Loeng, Edward Karam, Edward Lindisfarne, Edward Jenner, Elmunzar Bagouri, Ganapathy Raman Perianayagam, Gavin Schaller, George Mamarelis, George Matheron, Grahame Shaw, Greg Pickering, Guy Morris, Hammaad Khalil, Hean Wu Kang, Helen Vint, Huw Williams, Ian Cunningham, Islam Abdelrahman, Jagmeet Bhamra, Jagwant Singh, James Berwin, James Corbett, James Cruickshank, James Geddes, James Gill, James Pegrum, James Shelton, Jay Watson, Jermaine Thompson, Jimmy Ng, Joanna Higgins, John Davies, John Holgate, John Jackson, John Machin, John Vernon, John White, Jonathan Kent, Jonathan Quayle, Joseph Alsousou, Joseph Littlechild, Joseph Turner, Joshua Balogun-Lynch, Kamalpreet Cheema, Kathryn Kneale, Khabab Osman, Kim Shuttlewood, Laura Bolton, Laura Clifton, Liam Murphy, Liam Yapp, Lilanthi Wickramarachchi, Lily Li, Lucia Grossodi Palma, Luckshman Bevan, Lucy Maling, Luke Farrow, Mahdi Yacine Khalfaoui, Maire-Clare Killen, Malek Racy, Manav Raghuvanshi, Manish Divekar, Manish Kiran, Marcus Cope, Mark Higgins, Mark McMullen, Mark Webb, Marshall Sangster, Matilda Powell-Bowns, Michael Grant, Michael Petrie, Michalis Panteli, Mike Hogan, Moez Zeiton, Mohsen Raza, Muhammad Ahsan, Muhammad Adeel Akhtar, Mustafa Rashid, Nagriz Seyidova, Nancy Hadjievangelou, Naomi Gibbs, Natalia Kurek, Natasha Picardo, Nathan Campbell, Nathan Moore, Nick Kalson, Nickil Agni, Nicola Blucher, Nomaan Sheikh, Numan Shah, Oli Shastri, Onur Berber, Pamela Garcia Pulido, Parag Raval, Patrick Williams, Paul Cameron, Paul Haggis, Paul Hegarty, Paul Robinson, Payam Tarrossoli, Peter Cay, Peter Davies, Peter Smitham, Pinelopi Linardatou Novak, Piyush Mahaptra, Pranai Buddhev, Pranai Buddhev, Prashant Singh, Prithviraj Hallikeri, Rafik Fanous, Rajpal Nandra, Rakan Kabariti, Ramsay Refaie, Ramsey Chammaa, Ravi Gogna, Ravi Popat, Ray Chari, Richard Holleyman, Richard Limb, Roxanne Kulec, Rupert Wharton, Sabri Bleibleh, Sally-Anne Phillips, Sara Dorman, Saroosh Madanipour, Scott Muller, Scott Wilson, Shahrier Sarker, Sheraz Malik, Sheweidin Aziz, Shirley Lyle, Simon Fleming, Simon Humphry, Simond Jagernauth, Sinziana Contanstin Marino, Somashree Chatterji, Stefanie Andrew, Steve Kahane, Sunny Parikh, Surjit Lidder, Sushmith Ramakrishna, Syed Bokhari, Tarek Boutefnouchet, Thomas Knapper, Thomas Voller, Thomas Murphy, Tim Brock, Timothy Batten, Togay Koc, Toni Ardolino, Tony Antonios, Traian Vaidean, Tricia Walker, Vinesh Godhania, Vittoria Bucknall, Will Keiffer, William Chaundy, Zacharia Silk, Zain Sadozai, Zain Sohail, Mustafa S. Rashid

**Affiliations:** Botnar Research Centre, Windmill Road, Headington, Oxford, OX3 7LD England

**Keywords:** Surgeons, Training, Orthopaedic, Trauma, Exposure, Surgery

## Abstract

**Background:**

There are concerns regarding early years’ training for junior doctors in Trauma & Orthopaedic Surgery (T&O) in the United Kingdom. Our primary objective was to audit the clinical activities undertaken by junior doctors working in Trauma & Orthopaedic (T&O) surgery in the National Health Service (NHS) in a typical workweek. A secondary objective was to audit the clinical exposure of junior surgeons in training to the Joint Committee on Surgical Training (JCST) standards for minimum weekly clinical exposure in T&O surgery.

**Methods:**

We recruited collaborators in 101 T&O surgery departments in NHS hospitals to participate in this study. Clinical activity diaries from 935 doctors working in T&O surgery in the 101 participating NHS hospitals were involved. All junior doctors covering the junior on call tier were included. Collaborators collected clinical activity data from 08:00 18/01/2015 to 20:00 22/01/2015. Clinical activities recorded in sessions (morning, afternoon, evening) depending on what activity that doctor undertook for the majority of that session. Clinical activities were grouped into operating theatre/room, outpatient clinic, on call, “not in work” (i.e. leave, sickness), teaching, and ward cover sessions. The weekly clinical activity of Core Surgical Trainees (CSTs) were analyzed in accordance to two JCST standards for minimum weekly clinical exposure.

**Results:**

Overall, junior doctors working in T&O surgery attended a theatre list session 8.5% of the time, an outpatient clinic 3.2%, were on call 14.8%, a teaching session 1.7%, providing ward cover 34.6%, and on a zero session 20.7% of the time. Only 5% of core surgical trainees (*n* = 200) met both the JCST standards for minimum weekly clinical exposure in the specialty.

**Conclusions:**

Junior surgeons in training, working in Trauma & Orthopaedic surgery in the United Kingdom are not meeting the minimum weekly clinical sessions laid out by the JCST. Further work to develop models allowing for enhanced training experiences and improved clinical exposure to operating lists and outpatient clinics would be beneficial.

**Electronic supplementary material:**

The online version of this article (10.1186/s12909-017-1038-5) contains supplementary material, which is available to authorized users.

## Background

The latest reform to postgraduate medical training, Modernising Medical Careers (MMC) in 2005, brought about a new era in surgical education in the United Kingdom. Currently, medical graduates undertake a 2 year internship where they typically work in 6 posts of 4 months duration each. This is known as Foundation Training. One or two of these posts may be in a surgical specialty, including Trauma & Orthopaedic (T&O) surgery. During their Foundation Year 2 (FY2), doctors will apply to a Core Surgical Training programme (CST). Successful candidates will then undertake a 2 year programme, known as Core Surgical Training (CST) in an assigned geographical region. Core Surgical Training programmes vary in the number and duration of surgical posts. Typically, they will either be “generic”, including 3 or 4 specialties or “themed”, providing trainees with up to 18 months of training in a particular surgical discipline, e.g. Trauma & Orthopaedic surgery, out of 24 months. Posts are typically 4 or 6 months in duration, depending on the programme. During this 2 year training programme, junior surgeons follow a surgical curriculum, and are required to fulfil core competencies, as well as successfully undertake an examination, conducted by the Royal College of Surgeons. During their second year of Core Surgical Training (CT2), junior surgeons will apply for higher specialist training, often referred to a specialist registrar training, in a surgical discipline. There are 29 programmes in Trauma & Orthopaedic surgery in the United Kingdom. They are typically 6 years in duration. Specialist Training Registrars (StRs) are referred to as ST3 if in their first year, ST4 in their second year, and so on. The attainment of all competencies, including successfully passing a specialty exit examination, undertaken in ST7 or ST8, leads to an award of Certificate of Completion of Training (CCT).

Throughout surgical training in the United Kingdom, junior surgeons are expected to keep an online surgical logbook, and an electronic training portfolio. The electronic portfolio is a record of training competencies achieved. There are numerous assessments expected of junior surgeons, collectively known as Workplace-Based Assessments (WBAs), which are recorded by trainees and validated by consultant supervisors.

Workplace-based assessments (WBAs) were introduced in the United Kingdom in 2005 [Pitts 2005]. They have been expanded to include, multi-source feedback (MSF), directly observed procedural skills (DOPS), procedural based assessments (PBAs), case-based discussions (CBDs), and clinical evaluation exercises (CEXs). Surgeons in training are expected to complete a minimum of 40 or 80, dependant on training programme, in a single training year. These assessments are often undertaken during a clinical activity, such as an outpatient clinic or operating list, with a trained supervisor. Marriott et al. validated the use of PBAs to assess surgical competence across all surgical disciplines in the UK. They observed excellent construct validity, and high reliability. In this study, they state that amongst participants, there was good acceptability for the tool however only a small number of T&O surgical trainees were included [1]. Hunter et al. reported the trainees’ perspective on PBAs, demonstrating that of the 616 T&O surgeons-in-training completing their survey, just over half (53%) found them useful. They highlight the use of free text to record verbal constructive feedback as the most useful component of the assessment [2]. There is paucity of evidence relating to the application, or perceptions, of the other workplace-based assessments.

**Fig. 1:**
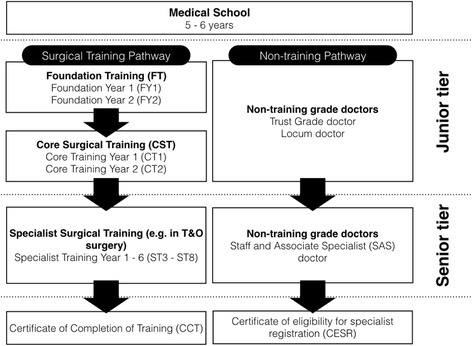
Flowchart outlines grades of doctor in postgraduate surgical training and non-training pathways in the United Kingdom

Postgraduate surgical training governance and outline is under the oversight of the Joint Committee on Surgical Training (JCST). This is made up of representatives from all 9 surgical specialties in the United Kingdom. The JCST produce guidelines and quality indicators (QIs) for each stage of surgical training, in each surgical discipline. These guidelines are to ensure that each surgical trainee is placed in a training post that provides sufficient training opportunities to achieve the required competencies outlined in the surgical curriculum. These QIs relate to Core Surgical Trainees and Specialist Trainees in all 9 surgical disciplines, i.e. those in a surgical training programme only. There are many grades and types of doctors working within surgery in the National Health Service (NHS) (Fig. 1). Doctors working in surgery outside a training programme may be Trust Grade doctors, i.e. employed by a particular hospital, or locum doctors, employed by an agency and work shifts as needed. Other doctors in non-training positions must fulfil criteria of professional development in order to maintain a medical license with the General Medical Council (GMC) however, they are not mandated to follow a curriculum or complete workplace-based assessments. There are other doctor grades that may work in the junior tier. Doctors undertaking a research post that includes some clinical duties are called Research Fellows (RFs). In Scotland, clinical development fellows (CDFs) are those wishing to secure a formal training post after gaining further experience. Occasionally in T&O, trainees in General Practice will work in the junior tier to gain experience of managing musculoskeletal conditions. In some hospitals, allied healthcare professionals, usually experienced nurses or physiotherapists, work alongside doctors in the junior tier, performing similar tasks. In some hospitals, first year specialist trainees (ST3s) work alongside other doctors in the junior tier, usually helping cover the on call duties.

Working hours’ restrictions for surgeons are applied in a number of countries around the world. The UK and the National Health Service (NHS), abides by the European Working Time Regulations (EWTR). These were introduced for doctors in 2009 and limit the hours worked in a typical week to an average of 48 h. In 2003, the United States introduced an 80-h weekly restriction for doctors. Baskies et al., analysed the logbooks of 109 Orthopaedic residents in New York, and demonstrated a reduction in operative volume due, after the introduction of working hours’ restrictions [3]. Froelich et al. demonstrated no significant difference in the average number of cases performed by Orthopaedic residents or the Orthopaedic In-Training Examination (OITE) scores after the introduction of working hours’ restrictions [4]. However, this study compared a smaller number of logbooks (35 residents pre-restrictions vs 62 post-restrictions), than the Baskies study, which may have influenced the findings. A systematic review, by Harris et al., on the effects of work-hour restrictions on education, quality of life, and safety concluded a paucity of research in this field [5]. To our knowledge, there are no studies published relating to clinical activities of junior doctors in T&O in the UK prior to EWTR introduction to allow comparison.

In a study following 4 orthopaedic surgery residents for 72 h in a major trauma centre in the United States, Hamid et al. reported proportions of time spent performing various clinical and nonclinical activities. They reported that these 4 trainees spent 26% of this time performing administrative tasks, 23% in direct contact with patients, 17% in communication with other healthcare professionals, and 16% on “non-productive work” [6]. Whilst this provides some insight into what these residents did during shifts, it is not translatable to trainee surgeons working in a number of different hospitals in the United Kingdom. Aside from the obvious differences in healthcare systems, experience levels of residents, and the variability between hospital workloads, this study cannot be extrapolated, as it is only 4 trainees in one hospital [6]. Similarly, in an audit of Core Surgical Trainee logbook experience of 9 trainee surgeons working in a major trauma centre in the United Kingdom, Morris et al. reported an increase in operative volume from 20.4 to 30 cases over a 4 month period after initiation of a new rota. It is commendable that this hospital improved surgical volume with a better rota design, however it does not allow us to appreciate what junior doctors working in T&O surgery are doing across a healthcare system, in many different hospitals [7]. This study does demonstrate that an improvement in clinical exposure for junior surgeons in training can be achieved by improving rota scheduling to emphasise activities that permit sufficient training opportunities.

There remains a paucity of evidence relating to the clinical exposure of junior surgeons in training, particularly differences between different national systems. One study conducted with Irish junior surgeons in training (*n* = 22) showed a perceived reduction in the development of operative skills (72%), and the quality of their training in general (88%) [8]. Maisonneuve et al. conducted a larger cross-specialty survey of junior doctors’ views toward EWTR impact on the NHS. They noted surgical respondents (*n* = 594), the majority believed working hours restrictions had a negative impact on the NHS (75.9%) and junior doctors (69.9%) [9].

Given the introduction of work-hours regulations, and a perceived reduction in training opportunities within Trauma & Orthopaedic surgery in the United Kingdom, we aimed to evaluate the clinical exposure of junior doctors working in T&O departments in the NHS. Specifically, we wished to audit the clinical activity of Core Surgical Trainees against the JCST Core Quality Indicators (QIs) [10].

This audit was conceived and designed by an elected committee of specialist training registrars from across the United Kingdom. This committee met on 4 occasions to determine which of the JCST Core Quality Indicators relating to Core Surgical Trainees working in T&O surgery are feasible to audit on a large scale (> 100 hospital sites). It was determined that JCST Core QI no. 10 and 16 met the criteria described [10]. Table 1 in the supplementary information outlines the reasons for inclusion / exclusion for all quality indicators relating to Core Surgical Trainees. A project was designed to audit clinical activity of junior doctors against these 2 standards, including a data collection spreadsheet, a how-to guide, and information pack.

**Table 1:** Clinical activity codes categorised into groups for final analysis

Theatre Sessions	Clinic	Off Work	Teaching	On Call	Ward Cover
Trauma theatre - TT	Fracture clinic - CF	Zero sessions - Z	Local teaching - LT	On call - OC	WC
Elective theatre - TE	Elective clinic - CE	Other leave - OL	Regional teaching - RT	Covering on call due to rota gap - CR	
		Annual leave - AL	Administrative / Research / Audit - AD	Covering on call due to unexpected sickness - CU	
		Study leave - SL	Multidisciplinary meeting / X-ray meeting / Other educational activity - MDT		

## Methods

### Coordination

This national multi-centre audit project was coordinated by the British Orthopaedic Trainees Association (BOTA) committee in association with the British Orthopaedic Network Environment (BONE).

### Recruitment

Collaborators were recruited between 12/09/2015 and 17/01/2016. Two hundred and forty-eight participants registered their interest during this time. The audit project was launched at the British Orthopaedic Association (BOA) Annual Congress 2015 in Liverpool to just under 2000 delegates. Throughout the recruitment period the project was advertised on the BOTA website with links for details and registration through BONE. One thousand and fifty-three BOTA members were contacted by email for participation and this was further publicised in person to those members attending Extraordinary General Meeting (EGM) on 10/01/2016. Thirty-three UK medical schools were contacted via university surgical and medical societies to enhance student involvement. All registrants were sent a welcome pack including a data collection sheet and project guide.

### Data collection

A data collection spreadsheet was designed by the project committee, made up of specialist training registrars, to allow simple and pragmatic collection of data across a large number of hospitals. Data was collected by participating collaborators, whom are surgeons in training in participating hospitals. Data collected on doctors working in the junior tier in Trauma & Orthopaedic surgery, and the clinical activity undertaken by each doctor during a typical workweek. It was not feasible for a local collaborator to accurately record hour-by-hour clinical activity diaries for up to 23 doctors working in the same hospital prospectively, hence, clinical activity was recorded in sessions. Junior doctors working in the NHS are contracted to work 08.00–17.00 when not on call. On calls in most hospitals are split between “daytime” i.e. 08.00–20.00 or “night time” i.e. 20.00–08.00. Hence, the working day was split into three sessions, “morning”, defined as 08.00–13.00, “afternoon/evening”, defined as 13.00–20.00, and “night”, defined as 20.00–08.00. Collaborators were instructed to record what clinical activity each doctor undertook “for the majority (>50%) of that session”. Although the sessions were of different durations, this does reflect working practices in the NHS, as there are minimal outpatient clinics beyond 17.00, and no regular operating lists run beyond 20.00. Clinical activity codes were described to incorporate the majority of those undertaken by junior doctors in the NHS including elective and trauma operating lists, fracture and elective orthopaedic clinics, educational activities such as attendance of regional teaching programme session, multidisciplinary team meetings, leave, and on call duties. These were pooled into broader categories to permit analysis and presentation. The categories included “Theatre Sessions”, “Clinics”, “Off work”, “Teaching”, “On Call”, and “Ward Cover”. Clinical activities included in each category in provided in Table 1.

Between Monday 18th January 08:00 and Friday 22nd January 20:00, data was collected prospectively from 228 collaborators in 101 NHS hospitals in the UK. Collaborators were all members of the British Orthopaedic Trainees Association, an organisation of junior doctors training in T&O surgery in the UK. Collaborators completed a coded clinical activity diary of all those that participate in the junior on call rota for Trauma & Orthopaedics in their hospital, traditionally called ‘Senior House Officers’ or ‘SHOs’.

Both the grade and clinical activities of each junior doctor on the junior tier rota were recorded in an encrypted Microsoft Excel spreadsheet ‘Data Collection Sheet’. Each day was broken up into three periods: AM, PM and Nights, with the corresponding clinical activity recorded for each period to reflect typical work schedules.

Clinical activity was coded using the ‘clinical activity key’ and grouped for the purposes of final analysis (Tables 1 and 2) as described above.

**Table 2:** Clinical activity codes categorised by training or non-training activity for final analysis. On call sessions are treated as a separate category

Included in “Training Activity”	Included in “Non-training Activity”	Excluded from both categories
Trauma theatre - TT	Zero sessions - Z	On call - OC
Elective theatre - TE	Ward cover - WC	Covering on call due to rota gap - CR
Fracture clinic - CF	Annual leave - AL	Covering on call due to unexpected sickness - CU
Elective clinic - CE	Study leave - SL	
Local teaching - LT	Other leave - OL	
Regional teaching - RT		
Multidisciplinary team meeting - MDT		
Administrative / Research / Audit - AD		

Collaborators that sent two files from the same department were asked to cross-tabulate their data for discrepancy and submit one file. Dual site trusts were asked to record a file for each site. Collaborators submitted their data within 7 days of the completion date for the audit (29th January 2016).

### Analysis

An analysis of activity included doctor grade, rota gaps, operative exposure, clinic exposure, on call activity, and ward cover, amongst others. Breakdown of doctor grade contributing to the junior on call rota were calculated along with percentage attendances to different environments such as elective or emergency theatres, and outpatient clinics.

Additionally, we collated clinical activities that carry a significant training component and compared the proportion of time spent in these “training activities” compared to those considered of little educational value, deemed “non-training activities”. The clinical codes included in each group are outlined in Table 2. On call duties can vary in their balance between training opportunities and service provision, hence these sessions were excluded from this training vs non-training activity analysis.

### Audit standards

Audit standards were ‘Quality Indicators’ for orthopaedic trainees outlined by The Joint Committee on Surgical Training (JCST) [10]. The reasons for inclusion and exclusion of Core QIs are outlined in Table 1 in the supplementary information. Due to the pragmatic nature and scale of this audit (> 100 hospitals), we used JCST Core QIs no.10 and 16. Quality indicator 10 relates to ‘weekly consultant supervised sessions’ of which a minimum of 5 sessions (with each session being more than 4 h) is the minimum standard. JCST Core QI 16 for T&O trainees specifically states “Core trainees in T&O should have the opportunity to attend three operating sessions (2× trauma and 1× elective) and at least one fracture clinic each week.”.

### Statistical analysis

**Fig. 2:**
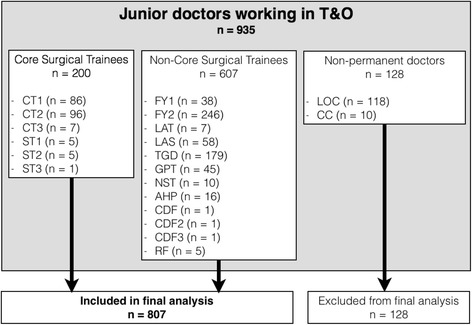
Flowchart demonstrating Cohort groups, split into Core surgical trainees, non-core surgical Trainees, and non-permanent doctors. CT1 = Core trainee year 1, CT2 = Core trainee year 2, CT3 = Core trainee year 3, ST1 = Specialist trainee year 1, ST2, = Specialist trainee year 2, ST3 = Specialist trainee year 3, FY1 = Foundation doctor year 1, FY2 = Foundation doctor year 2, LAT = Locum appointment for training, LAS = Locum appointment for service, TGD = Trust grade doctor, GPT = General practice trainee, NST = Other non-surgical trainee, AHP = Allied healthcare professional, CDF = Clinical development fellow, RF = Research fellow, LOC = Locum doctor, CC = Cross-cover doctor

Permanent staff working in the T&O department were included in the final analysis, excluding those doctors providing cross-cover from other specialities for on-call sessions, and locum doctors whom work a variable number of sessions (Fig. 2). Core Surgical Trainees’ clinical activity categories was compared to non-core surgical trainees in two cohort groups (Table 2). Median, 10th and 90th centile values were used for descriptive statistics due to the skewness of this non-parametric data. Using SPSS v22 software (IBM, Armonk, USA), independent samples median test was used to determine statistical significance between these two groups for each clinical activity category. For comparison between CST doctors and non-CST doctors, regarding training versus non-training activities, Chi-squared test was used. A significance level of <0.05 was set.

## Results

**Fig. 3:**
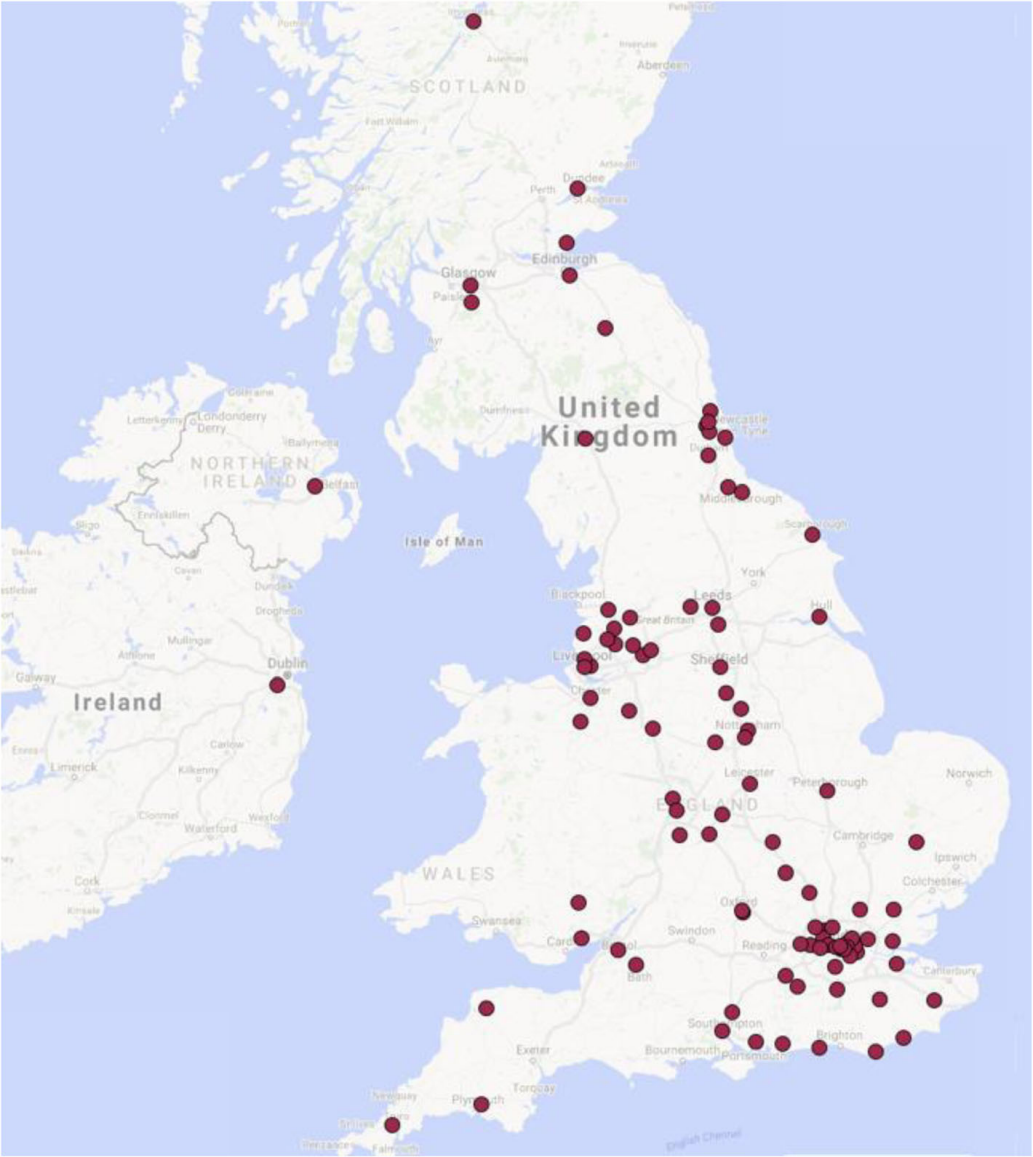
Map of United Kingdom and Ireland demonstrating geographical spread of the 101 participating hospitals

One hundred and one hospitals in the National Health Service (NHS) in the United Kingdom participated in the study. There are 152 acute NHS trusts in the United Kingdom [11]. A map of participating hospitals is provided (Fig. 3) and demonstrates the breadth of participating sites geographically. In these hospitals, there were 935 junior doctors in the most junior tier (i.e. junior tier or formerly, Senior House Officer (SHO) grade) in Trauma and Orthopaedic surgery. The mean number of doctors at this level per department was 9 (range 1–23). In the 101 hospitals that participated, there were 32 documented rota gaps. The breakdown of grades of doctors is illustrated in Fig. 2. Core surgical trainees made up 21.6% of the cohort (*n* = 200) with a mean of 1.9 CSTs per T&O department (range 0–5). For the purposes of further analysis, we excluded those doctors who provided cross-cover from other specialties as well as locum doctors (*n* = 128). This left 807 doctors for final analysis.

**Fig. 4:**
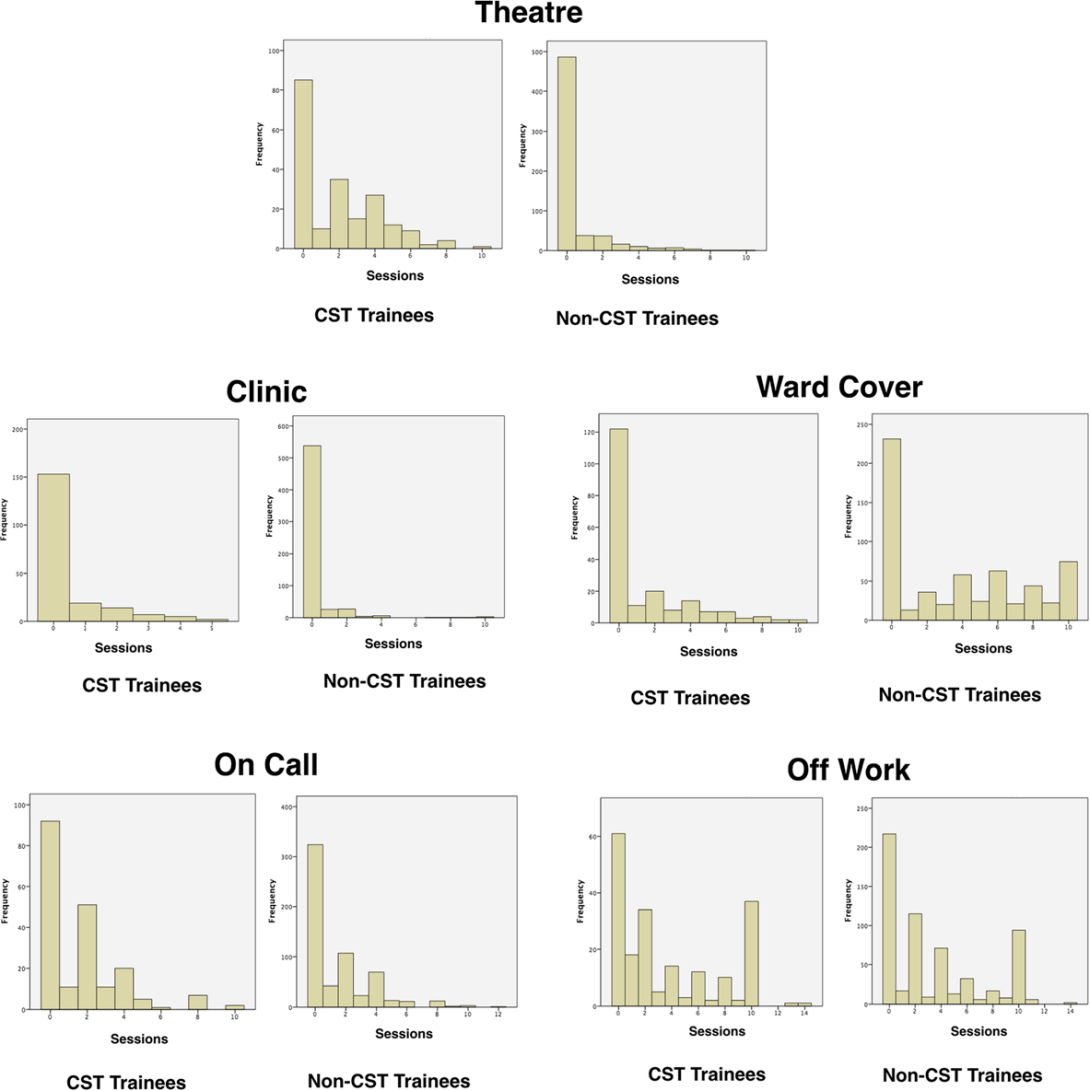
Histograms illustrating frequency of number of sessions undertaken by Core surgical trainees versus non-Core surgical trainees in Theatre, Clinic, Ward Cover, On Call, and Off Work

The clinical activities recorded in the data collection spreadsheet was designed to be exhaustive, covering the vast majority of activities. The surgical training curriculum however, identifies theatre sessions, outpatient clinics, on call duties, structured teaching as being relevant for trainees acquiring core competencies. After pooling clinical activity codes (as per Table 1), we conducted an analysis of difference in types of activities undertaken between Core Surgical Trainees and Non-Core Surgical Trainees. The categories evaluated were “Off Work”, “Theatre”, “Clinic”, “On Call”, “Ward Cover”, and “Teaching”. “Off work” and “Ward cover” were included as trainees performing these activities will, by definition, not be attending the other activities and therefore unable to achieve core competencies during these sessions. The data was found to be non-parametric (Fig. 4). The median as well as 10th/90th centiles for each category are shown in Table 3 for both CST and non-CST cohort groups.

**Table 3:** Median number of sessions, 10th and 90th centiles (in brackets) of number of sessions undertaken by Core Surgical Trainees, and non-Core Surgical Trainee doctors in off work, theatre, clinic, on call, ward cover, and teaching session categories. Non-parametric independent samples median test with *p* values shown

	CST Trainees (n = 200)	Non-CST Trainees (*n* = 607)	Independent samples median test
	Median number of sessions	Median number of sessions	Significance (p value)
	(10th - 90th Centiles)	(10th - 90th Centiles)	
Off Work	2 (0–10)	2 (0–10)	0.869
Theatre	2 (0–5)	0 (0–2)	**<0.001**
Clinic	0 (0–2)	0 (0–1)	**<0.001**
On Call	1 (0–4)	0 (0–4)	0.084
Ward Cover	0 (0–5)	4 (0–10)	**<0.001**
Teaching	0 (0–2)	0 (0–1)	**<0.001**

The bold *p* values shown are meant to represent those that met the stated level of significance of < 0.05

Non-parametric statistical analysis was performed to determine a difference between the medians for each category. Despite seemingly little or no difference in median number of clinics, theatre sessions, and teaching sessions attended, they were determined to be statistically significantly different. This was due to the small number of doctors in some hospitals skewing the data to the right, i.e. undertaking a significantly larger number of clinics, theatre sessions and teaching sessions. There was no significance between number of on call sessions, and number of sessions “Off Work”. Core Surgical Trainees did perform less Ward Cover sessions than their non-trainee colleagues. (Median 0 vs 4, *p* < 0.001).

**Fig. 5:**
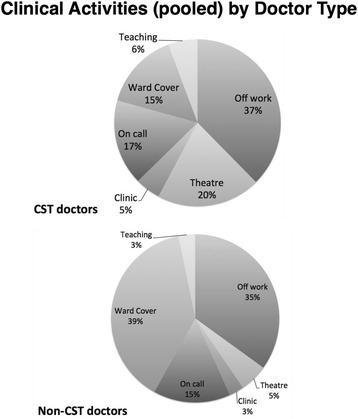
illustrates the proportions of clinical activities undertaken by Core Surgical Trainees and non-Core Surgical Trainee doctors

When looking simply at the proportions of each clinical activity category by cohort, we can see clear differences in work schedules (Fig. 5). Core Surgical trainees did a higher proportion of operating theatre sessions, on call sessions, outpatient clinics, and teaching. Non-core surgical trainees performed more ward cover sessions than Core Surgical Trainees.

### JCST core quality indicators

**Fig. 6:**
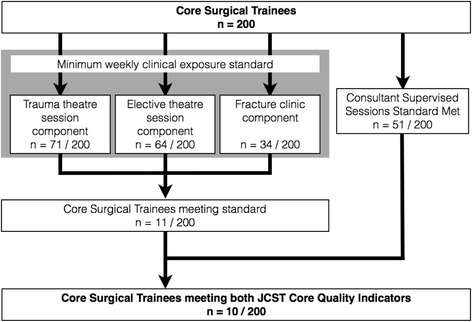
Flowchart demonstrating numbers of Core surgical trainees meeting the two national JCST Core Quality Indictors relating to T&O surgery

Two national standards, set out by the JCST committee, related to Core Surgical Trainees were audited against. The first standard states that a Core Surgical Trainee must undertake a minimum of five consultant-supervised sessions (with each session being more than 4 h) each week. Fifty-one out of two hundred (25.5%) CSTs met this criterion during the study period. The second standard relates specifically to CSTs working in T&O, and states that CSTs should undertake a minimum of two trauma operating session, one elective operating session, and one fracture clinic each week. In total 11 out of 200 (5.5%) CSTs met this criterion (a detailed breakdown of the components of this standard and numbers of CSTs meeting each component is shown in Fig. 6). Additionally, when combined, only 10 out of 200 (5%) CSTs in T&O in 101 participating hospitals met both of these 2 national standards for clinical exposure and training.

### Training versus non-training activities

The proportion of training and non-training activities was also evaluated for the two different cohort groups. Core Surgical Trainees spent 16% of their workweek on call, 31% undertaking a primarily training-focussed activity, and 53% undertaking non-training activities. In contrast, non-Core Surgical Trainee doctors undertook 14% on call sessions, 12% training activities, and 74% non-training activities. Whilst proportion of on call sessions between these two groups was not statistically significant, CST doctors undertook more training-related activities (*p* < 0.0001).

## Discussion

This is the largest trainee led audit in the UK evaluating training opportunities for junior surgeons. This study examined the clinical activities undertaken by a junior tier of doctors working in Trauma & Orthopaedic Surgery departments across one hundred and one hospitals. We have demonstrated that Core Surgical Trainees, at the beginning of a long surgical career, are undertaking different proportions of clinical activities to other doctors, not undertaking a postgraduate surgical training programme. Principally, they attend more operating theatre sessions, clinic sessions, and teaching sessions. They do less ward cover sessions than their non-training colleagues, and the same number of on call sessions and off work sessions. Despite working seemingly different work schedules, Core Surgical Trainees rarely met the two national standards on clinical exposure and training we audited. Only 10 out 200 (5%) Core Surgical Trainees in T&O met the JCST Core Quality Indictors during a typical workweek.

In the United Kingdom, doctors completing foundation training wishing to pursue a surgical career enter Core Surgical Training. They spend 24 months following a broad and detailed surgical curriculum, attaining core competencies in the generality of surgery. They undertake clinical activities that allow demonstrating of surgical skill and knowledge through a number of workplace-based assessments, recorded in a training portfolio, as well as an electronic logbook of surgical experience. To ensure each training post is fit for training, the organisation responsible for oversight in postgraduate surgical training, the JCST, outlines Core Quality Indicators for all CST doctors. Many of these relate to the collective experience over a post’s duration (typically 4 or 6 months), but two relate to weekly clinical exposure and so were chosen for this pragmatic and large-scale audit. It is concerning that these minimum standards for clinical exposure were not met by 95% of Core Surgical Trainees during a typical workweek.

In order to allow Core Surgical Trainees to undertake the required clinical sessions to follow the surgical curriculum and progress in their training, hospitals may consider the use of allied healthcare professionals (AHPs). In the NHS, this may include nurse practitioners, extended scope physiotherapists, and physician associates. Physician associates in North America have been shown to integrate well in arthroplasty services [12]. They have been shown to save physicians’ time by performing simple tasks such as writing discharge summaries, medication charts, and requesting investigations [12]. Another study demonstrated that they are able to perform more complex tasks such as fracture reductions and casting in paediatric fractures, comparable to residents in training [13]. In the US model, it costs 25% the cost of training an Orthopaedic resident to train a physician associate in a 2.5-year postgraduate Masters programme. In 2013, there were over 89,000 licensed PAs working in US healthcare [14]. However, a balance must be carefully considered to ensure AHPs working in surgery enhance training opportunities for junior surgeons in training, rather than replace their role.

Another means of ensuring Core Surgical Trainees achieve minimum clinical exposure in T&O, as outlined by the JCST core quality indicators, hospital department should consider protecting work schedules for this group of doctors. There are activities, which are of perceived low educational value, such as ward cover, which often involves writing discharge summaries, rewriting medication charts, and organising radiological investigations. The other 80% of the workforce at this level can potentially cover these responsibilities, freeing up Core Surgical Trainees to attend theatre and clinic sessions. A change in rota scheduling for Core Surgical Trainees demonstrated significant increase in training opportunities in a study conducted at Queen’s Medical Centre, Nottingham, UK [7]. An even more radical solution is to consider whether CSTs should undertake night shifts on call at all. These have some educational value as well as a significant service delivery component. However, in order for rotas to be compliant with European Working Time Regulations (EWTR) [15], 11 h of rest following a shift must be factored in. Periods of rest, or “Zero Sessions”, during the day, which useful educational opportunities most commonly occur, leads to missed training opportunities. The CST cohort is sufficiently small (20% and a mean of 1.9 trainees per department), that producing a different on call shift pattern for them may be a feasible way to ensure more training opportunities during the week. There are however, significant cultural, financial, and practical challenges that this faces.

This study has several limitations including the effect of rotating work schedules, and the degree of granularity of data collection. It is possible, that in a given hospital, on this typical working week, the Core Surgical Trainees in that department were on call, leading to little, or no, theatre or clinic exposure. This however, is mitigated for the large number of participating hospitals (*n* = 101) that provide a good representation of the NHS for a typical week. Given that 935 doctors’ clinical activities were monitored over 101 hospitals, the effect of rotating scheduling is minimised.

Another limitation relates to data collection. In order to reduce the burden on collaborators, and to ensure accurate data collection in a simple and pragmatic manner, we reduced the degree of granularity to three sessions per day, which reflect the clinical activity sessions in the NHS. Collaborators were asked to record what each doctor did for the majority of that session. This lack of hour by hour recording of data collection is a limitation because a doctor undertaking a trauma meeting for an hour before being on ward cover the rest of the day would be recorded as ward cover for the morning and afternoon sessions. It is worth noting that the JCST Core quality indicator for the number of consultant supervised sessions states a session must be a minimum of 4 h, so it is felt that this compromise is justified as it matches the requirements of the national standard we audited against.

The findings of this study provide some interesting observations that should be explored in future work. Specifically, this audit did not attempt to evaluate the quality of the training opportunities provided to Core Surgical Trainees. Additionally, how does the number of training opportunities relate to a successful career in T&O surgery? What impact does the current state of T&O training for junior doctors in the NHS have on patient outcomes? As an audit of training standards for Core Surgical Trainees, we identified that the current standards for minimum weekly clinical exposure are not being met. Hence, a strategy for improving compliance must be drawn up in each hospital, implemented appropriately, and a repeat audit conducted to evaluate the effect of any changes.

## Conclusions

Despite junior surgeons in training undertaking more teaching, operating, and outpatient clinic sessions than colleagues not in a training programme, they are not meeting the minimum national standards for clinical exposure in Trauma & Orthopaedic surgery in the United Kingdom. As a profession, we need to do more to ensure these doctors are able to develop as junior surgeons and follow the surgical curriculum, attain core competencies, and progress through their training. Further work to identify reasons for limited training exposure, and to research methods to increase clinical exposure for junior surgeons in training working in T&O must be a priority for the future.

## Additional file

Additional file 1: A list of all co-authors and participating hospital sites. (DOCX 29 kb)

## Abbreviations

AD: Administrative session
AL: Annual Leave
BMA: British Medical Association
BOA: British Orthopaedic Association
BONE: British Orthopaedic Network Environment
BOTA: British Orthopaedic Trainees Association
CE: Elective Outpatient Clinic
CF: Fracture clinic
CR: Covering on call due to rota gap
CST: Core Surgical Training
CU: Covering on call due to unexpected sickness
EGM: Extraordinary General Meeting
EWTR: European Working Time Regulation
HST: Higher specialist training
JCST: Joint Committee for Surgical Training
LT: Local teaching
MDT: Multi-disciplinary team
MMC: Modernising Medical Careers
NHS: National Health Service
NTN: National Training Number
OC: On call
OL: Other leave
RT: Regional teaching
SHO: Senior house office
SL: Study leave
SpR: Specialist registrar
StR: Specialist training registrar
T&O: Trauma and Orthopaedic Surgery
TE: Elective theatre
TT: Trauma theatre
WC: Ward cover
Z: Zero session
