# Correction to: An audit of clinical training exposure amongst junior doctors working in Trauma & Orthopaedic Surgery in 101 hospitals in the United Kingdom

**DOI:** 10.1186/s12909-018-1132-3

**Published:** 2018-02-19

**Authors:** BOTA Collaborators, Mustafa S. Rashid

**Affiliations:** Botnar Research Centre, Windmill Road, Headington, Oxford, OX3 7LD England

## Correction

Following publication of the original article [1], the corresponding author wrote to say that he had missed the names of some of the collaborators in the list he sent to the typesetters. In addition, there was a spelling error in one of the author’s names: instead of Nagriz Seyidova it should read Nargiz Seyidova. The complete list of collaborators is as follows:

Mustafa Rashid, Simon Fleming, Steve Kahane, Sara Dorman, Danny Ryan, James Shelton, Oli Shastri, Marshall Sangster, Helen Vint, Vittoria Bucknall, Paul Hegarty, Rupert Wharton, John Davies, Payam Tarassoli, and Peter Smitham, Ashley Scrimshire, Zain Sadozai, Aurelie Hay-David, Kim Shuttlewood, Huw Williams, Lucy Maling, Liam Murphy, Manish Divekar, Laura Bolton, Conal Quah, John Machin, Abdul Nazeer Moideen, Alexandra Aframian, Toni Ardolino, Sally-Anne Phillips, Matilda Powell-Bowns, James Geddes, Oli Shastri, Jonathan Quayle, Thomas Murphy, Greg Pickering, David Milligan, Hean Wu Kang, Jermaine Thompson, Ramsay Refaie, Nickil Agni, Mahdi Yacine Khalfaoui, Muhammad Ahsan, Hammaad Khalil, Basil Budair, Damian Bull, Thomas Knapper, John Jackson, Marcus Cope, Pinelopi Linardatou Novak, Lily Li, Daniel Burchette, Alisdair Felstead, Daniel Wilson, Blair Tweedie, Ali Abdulkarim, Muhammad Adeel Akhtar, Joseph Alsousou, Alexander Martin, Dan Williams, Anna Bridgens, Natasha Picardo, Sheraz Malik, Alex Mulligan, Gavin Schaller, Stefanie Andrew, Christopher Buckle, Barry Rose, David Butt, Nicola Blucher, Parag Raval, Jagmeet Bhamra, Amit Patel, Prashant Singh, Saroosh Madanipour, Patrick Williams, Mark McMullen, Mark Webb, Maire-Clare Killen, Sushmith Ramakrishna, Lucia Grossodi Palma, Traian Vaidean, Rajpal Nandra, Edward Jenner, Ravi Gogna, Mohsen Raza, Piyush Mahaptra, Alexander Durst, Alan Campbell, James Gill, Peter Cay, Paul Haggis, Islam Abdelrahman, Khabab Osman, Dan Howgate, Luckshman Bevan, Shirley Lyle, Natalia Kurek, Surjit Lidder, Amit Thakrar, John Holgate, Kamalpreet Cheema, Nancy Hadjievangelou, Sinziana Contanstin Marino, Edward Lindisfarne, Thomas Voller, Claire Coles.
