# Supplementary material for: An audit of clinical training exposure amongst junior doctors working in Trauma & Orthopaedic Surgery in 101 hospitals in the United Kingdom
Source: BMC Med Educ. 2018 Jan 2;18:1. doi: 10.1186/s12909-017-1038-5 (PMC5749005; doi:10.1186/s12909-017-1038-5)
Supplement: Additional file 1: — A list of all co-authors and participating hospital sites. (DOCX 29 kb) [file 12909_2017_1038_MOESM1_ESM.docx]

**List of co-authors and participating institutions**

**Study Steering Committee members:**

| Mustafa | Rashid |
| --- | --- |
| Simon | Fleming |
| Sara | Dorman |
| Steve | Kahane |
| Danny | Ryan |
| Marshall | Sangster |
| Rupert | Wharton |
| Paul | Hegarty |
| Payam | Tarrossoli |
| James | Shelton |
| Vittoria | Bucknall |
| John | Davies |
| Oli | Shastri |
| Peter | Smitham |
| Helen | Vint |

**Study collaborating authors:**

| **Hospital Name** | **Firstname** | **Surname** |
| --- | --- | --- |
| Aintree University Hospital | Michael | Grant |
| Alder Hey Children's NHS Foundation Trust | Manish | Kiran |
| Alexandra Hospital | Tarek | Boutefnouchet |
|  | Guy | Morris |
| Barnet Hospital | Jagwant | Singh |
|  | Simond | Jagernauth |
| Basildon University Hospital | Sunny | Parikh |
| Borders General Hospital | Liam | Yapp |
| Bradford Royal Infirmary | Ashwanth | Ramesh |
|  | Adedeji | Akinyooye |
| Broomfield Hospital | Shahrier | Sarker |
|  | Manav | Raghuvanshi |
| Central Middlesex Hospital | Ravi | Popat |
|  | Rafik | Fanous |
|  | Annie | McKirdy |
|  | Ed | Loeng |
| Chesterfield Royal Hospital | Malek | Racy |
| Chorley and South Ribble Hospital | Charlotte | Cross |
| Conquest Hospital | Christopher | Crome |
|  | Will | Keiffer |
|  | Paul | Cameron |
| Countess Of Chester Hospital | James | Shelton |
| Cumberland Infirmary | Jonathan | Kent |
| Derriford Hospital | Timothy | Batten |
|  | Kathryn | Kneale |
| Ealing Hospital | Charlotte | Richardson |
|  | Rupert | Wharton |
| East Surrey Hospital | Jay | Watson |
|  | Grahame | Shaw |
| Eastbourne District General Hospital | Tricia | Walker |
| Freeman Hospital | Andrea | Pujol Nicolas |
|  | Helen | Vint |
| Frimley Park Hospital NHS Foundation Trust | William | Chaundy |
| Glasgow Royal Infirmary | Donald | Hansom |
| Guy's Hospital | James | Berwin |
|  | Onur | Berber |
|  | Pranai | Buddhev |
|  | Joseph | Turner |
|  | Dinnish | Baskaran |
|  | Dev | Thakker |
|  | Lilanthi | Wickramarachchi |
| Hairmyres Hospital | Ian | Cunningham |
| Hillingdon Hospital | Daniel | Shaerf |
| Homerton University Hospital | Ali | Abdelwahab |
| Hull Royal Infirmary | Vinesh | Godhania |
|  | Numan | Shah |
|  | Syed | Bokhari |
| John Radcliffe Hospital | Laura | Clifton |
|  | Nagriz | Seyidova |
| King's Mill Hospital | Edward | Karam |
|  | John | Vernon |
|  | Prithviraj | Hallikeri |
|  | Richard | Limb |
|  | George | Matheron |
|  | Mark | Higgins |
|  | Ray | Chari |
| Leeds General Infirmary | Michalis | Panteli |
| Leicester Royal Infirmary | Sheweidin | Aziz |
|  | Ganapathy Raman | Perianayagam |
| Leighton Hospital | Sara | Dorman |
| Luton and Dunstable Hospital | Pamela | Garcia Pulido |
|  | Bhavin | Garara |
| Medway Maritime Hospital | Sabri | Bleibleh |
| Milton Keynes Hospital | Joshua | Balogun-Lynch |
|  | Andrew | Hacker |
|  | Aman | Sharma |
| Nevill Hall Hospital | Simon | Humphry |
|  | Rakan | Kabariti |
|  | Alexander | James |
| Newham University Hospital | Ramsey | Chammaa |
|  | Anoop | Prasad |
|  | Mike | Hogan |
| Ninewells Hospital and Medical School | Alistair | Mayne |
| North Devon District Hospital | Alex | Goubran |
| North Manchester General Hospital | Antonia | Hoyle |
|  | Scott | Wilson |
| Northampton General Hospital | Nomaan | Sheikh |
| Northern General Hospital | Michael | Petrie |
|  | Roxanne | Kulec |
| Northumbria Specialist Emergency Care Hospital | Tim | Brock |
|  | Nick | Kalson |
|  | Richard | Holleyman |
|  | Scott | Muller |
| Northwick Park Hospital | Rafik | Fanous |
|  | Annie | McKirdy |
|  | Ravi | Popat |
|  | Ed | Loeng |
| Nottingham City Hospital | Jimmy | Ng |
| Nuffield Orthopaedic Centre | James | Pegrum |
|  | Naomi | Gibbs |
| Peterborough City Hospital | Aaron | Rooney |
|  | James | Corbett |
| Pinderfields General Hospital | James | Cruickshank |
|  | Elmunzar | Bagouri |
| Princess Alexandra Hospital | Zacharia | Silk |
|  | Cameron | Dott |
|  | George | Mamarelis |
|  | Zain | Sohail |
| Queen Alexandra Hospital | Joanna | Higgins |
|  | Christopher | Jordan |
|  | Togay | Koc |
| Queen Elizabeth Hospital (Gateshead) | Christopher | Ghazala |
|  | Colin | Shaw |
| Queen Elizabeth Hospital (Woolwich) | Anatole | Wiik |
|  | Tony | Antonios |
| Queen's Hospital (Romford) | John | White |
|  | Amanjeet | Dahaley |
| Queen's Medical Centre | Nathan | Moore |
|  | Simon | Fleming |
|  | Sheraz | Malik |
| Raigmore Hospital | David | Neilly |
|  | David | MacDonald |
|  | Joseph | Littlechild |
|  | Luke | Farrow |
|  | Peter | Davies |
| Royal Albert Edward Infirmary | Ahmed | Fadulemola |
|  | Akmal | Turaev |
|  | Paul | Robinson |
| Royal Blackburn Hospital | Nathan | Campbell |
|  | Somashree | Chatterji |
|  | Moez | Zeiton |
|  | Ashley | Scrimshire |
| Royal Bolton Hospital | Zain | Sadozai |
| Royal Cornwall Hospital | Aurelie | Hay-David |
|  | Kim | Shuttlewood |
|  | Huw | Williams |
|  | Lucy | Maling |
|  | Liam | Murphy |
|  | Manish | Divekar |
| Royal Derby Hospital | Laura | Bolton |
|  | Conal | Quah |
|  | John | Machin |
| Royal Gwent Hospital | Abdul Nazeer | Moideen |
| Royal Hampshire County Hospital | Alexandra | Aframian |
|  | Toni | Ardolino |
| Royal Infirmary of Edinburgh | Sally-Anne | Phillips |
|  | Matilda | Powell-Bowns |
| Royal Preston Hospital | Charlotte | Cross |
| Royal Stoke University Hospital | James | Geddes |
|  | Oli | Shastri |
| Royal Surrey County Hospital | Jonathan | Quayle |
| Royal United Hospital | Thomas | Murphy |
|  | Greg | Pickering |
| Royal Victoria Hospital (Belfast) | Paul | Hegarty |
|  | David | Milligan |
|  | Hean Wu | Kang |
| Royal Victoria Infirmary | Jermaine | Thompson |
|  | Ramsay | Refaie |
|  | Nickil | Agni |
| Salford Royal NHS Foundation Trust | Mahdi Yacine | Khalfaoui |
|  | Muhammad | Ahsan |
|  | Hammaad | Khalil |
| Sandwell General Hospital | Basil | Budair |
| Scarborough General Hospital | Damian | Bull |
| Southmead Hospital | Thomas | Knapper |
|  | John | Jackson |
| Southport & Ormskirk Hospital NHS Trust | Marcus | Cope |
| St Helier Hospital | Pinelopi | Linardatou Novak |
| St Mary's Hospital, Imperial College | Lily | Li |
| St Richard's Hospital | Daniel | Burchette |
|  | Alisdair | Felstead |
|  | Daniel | Wilson |
| St Thomas' Hospital | James | Berwin |
|  | Onur | Berber |
|  | Pranai | Buddhev |
|  | Joseph | Turner |
|  | Dinnish | Baskaran |
|  | Dev | Thakker |
|  | Lilanthi | Wickramarachchi |
| Sunderland Royal Hospital | Blair | Tweedie |
| The Adelaide and Meath Hospital, Dublin | Ali | Abdulkarim |
| The James Cook University Hospital | Muhammad Adeel | Akhtar |
| The Royal Liverpool University Hospital | Joseph | Alsousou |
| The Royal London Hospital | Alexander | Martin |
|  | Dan | Williams |
|  | Anna | Bridgens |
|  | Natasha | Picardo |
|  | Sheraz | Malik |
| The Royal National Orthopaedic Hospital | Steve | Kahane |
|  | Alex | Mulligan |
|  | Gavin | Schaller |
|  | Stefanie | Andrew |
| Tunbridge Wells Hospital | Christopher | Buckle |
|  | Barry | Rose |
|  | David | Butt |
| University College London Hospital | Nicola | Blucher |
| University Hospital Coventry & Warwickshire | Parag | Raval |
| University Hospital Lewisham | Jagmeet | Bhamra |
|  | Amit | Patel |
|  | Prashant | Singh |
|  | Saroosh | Madanipour |
| University Hospital of North Durham | Patrick | Williams |
|  | Mark | McMullen |
|  | Mark | Webb |
| University Hospital of North Tees | Maire-Clare | Killen |
| University Hospital Southampton | Sushmith | Ramakrishna |
|  | Lucia | Grossodi Palma |
|  | Traian | Vaidean |
| University Hospitals Birmingham NHS Trust | Rajpal | Nandra |
| Victoria Hospital | Vittoria | Bucknall |
| Warwick Hospital | Edward | Jenner |
|  | Ravi | Gogna |
| Watford General Hospital | Mohsen | Raza |
|  | Piyush | Mahaptra |
| West Suffolk Hospital | Alexander | Durst |
|  | Alan | Campbell |
|  | James | Gill |
|  | Peter | Cay |
| Wexham Park Hospital | Paul | Haggis |
|  | Islam | Abdelrahman |
|  | Khabab | Osman |
|  | Dan | Howgate |
|  | Luckshman | Bevan |
| Whipps Cross University Hospital | Shirley | Lyle |
|  | Natalia | Kurek |
| William Harvey Hospital (Ashford) | Surjit | Lidder |
|  | Amit | Thakrar |
|  | John | Holgate |
|  | Kamalpreet | Cheema |
|  | Nancy | Hadjievangelou |
|  | Sinziana | Contanstin Marino |
| Worthing Hospital | Edward | Lindisfarne |
|  | Thomas | Voller |
| Wrexham Maelor Hospital | Claire | Coles |
| Wrightington Hospital | Ahmed | Fadulemola |
|  | Akmal | Turaev |
|  | Paul | Robinson |
